# Hydrogel-Based Depot Systems in Psychiatric Pharmacotherapy: A Narrative Review Rethinking Long-Acting Drug Delivery

**DOI:** 10.3390/gels12090855

**Published:** 2026-09-20

**Authors:** Emilia Denisa Predoi, Anca Târtea, Oana Taisescu, Alexandra Daniela Rotaru-Zăvăleanu, Ana-Maria Ifrim-Predoi, Roxana Costina Vlad, Bogdan Cătălin, Mădălina Iuliana Mușat, Manuel-Ovidiu Amzoiu, Andrei Greșiță

**Affiliations:** 1 Experimental Research Centre for Normal and Pathological Aging, University of Medicine and Pharmacy of Craiova, 2-4 Petru Rares Street, 200349 Craiova, Romania; dennissa_predoi@yahoo.com (E.D.P.); andrei.gresita@umfcv.ro (A.G.); 2 Department of Neurology, University of Medicine and Pharmacy of Craiova, 2-4 Petru Rares Street, 200349 Craiova, Romania; anca.tartea@umfcv.ro; 3 Department of Human Anatomy, University of Medicine and Pharmacy of Craiova, 2-4 Petru Rares Street, 200349 Craiova, Romania; oana.taisescu@umfcv.ro; 4 Department of Epidemiology, University of Medicine and Pharmacy of Craiova, 2-4 Petru Rares Street, 200349 Craiova, Romania; alexandra.rotaru@umfcv.ro; 5 Department of Surgery, University of Medicine and Pharmacy of Craiova, 2-4 Petru Rares Street, 200349 Craiova, Romania; anamaria.ifrim@umfcv.ro; 6 Department of Psychology, Faculty of Legal, Economic and Administrative Sciences, Spiru Haret University, Vasile Conta, no. 4, 200580 Craiova, Romania; roxana.vlad@spiruharet.ro; 7 Department of Physiology, University of Medicine and Pharmacy of Craiova, 2-4 Petru Rares Street, 200349 Craiova, Romania; 8 Department of Scientific Research Methodology, University of Medicine and Pharmacy of Craiova, 2-4 Petru Rares Street, 200349 Craiova, Romania; 9 Faculty of Pharmacy, University of Medicine and Pharmacy of Craiova, 200638 Craiova, Romania; manuel.amzoiu@umfcv.ro

**Keywords:** injectable hydrogels, depot drug delivery, long-acting injectables, antipsychotics, psychiatric pharmacotherapy

## Abstract

Poor adherence to pharmacotherapy remains a major challenge in the long-term management of severe psychiatric disorders. Although long-acting injectable (LAI) antipsychotics can provide effective and sustained drug exposure while supporting treatment continuity, their pharmaceutical and clinical characteristics vary considerably according to the active pharmaceutical ingredient and formulation technology. Specific formulations may be associated with limitations such as complex release kinetics, injection-site reactions, or the need for oral supplementation during treatment initiation. Hydrogel-based delivery systems have emerged as a versatile class of biomaterials that may address these limitations through their tunable physicochemical properties, high water content, biocompatibility, and capacity for controlled, sustained drug release. Their adaptable architecture further enables diverse drug-loading strategies and multiple routes of administration, expanding the design space for next-generation long-acting formulations. In this narrative review, we provide a comprehensive overview of hydrogel-based depot systems for psychiatric pharmacotherapy, focusing on hydrogel composition (natural, synthetic, and hybrid polymers), crosslinking mechanisms, stimuli-responsive and injectable formulations, drug loading and release strategies, and their translational potential. Current research has primarily focused on antipsychotic delivery, where hydrogel formulations of risperidone, paliperidone, aripiprazole, olanzapine, quetiapine and the investigational peptide PAOPA show possible sustained drug release and prolonged therapeutic activity in preclinical models. Compared with conventional depot technologies, hydrogel systems may offer greater formulation flexibility and the potential for more controlled pharmacokinetic profiles and prolonged drug exposure. These characteristics could ultimately support reduced dosing frequency and treatment continuity. We also discuss the key challenges limiting clinical translation, including optimization of release kinetics, scalable manufacturing, sterilization, long-term stability, and regulatory considerations. Collectively, current evidence supports the technological potential of hydrogel-based systems to provide tunable drug loading, depot formation, and sustained release. However, their development as clinically meaningful next-generation LAI therapies will require long-term in vivo evaluation, robust pharmacokinetic–pharmacodynamic characterization, injection-site safety assessment, reproducible and scalable manufacturing, and well-designed clinical trials.

## 1. Background

Severe psychiatric disorders, particularly schizophrenia and related psychotic disorders, require prolonged pharmacological treatment to reduce the risk of symptomatic exacerbation and relapse [1,2]. Antipsychotic medication remains a central component of maintenance treatment, but the effectiveness of long-term pharmacotherapy depends not only on the intrinsic efficacy of the active pharmaceutical ingredient but also on the ability to maintain treatment continuity over time [3,4]. In routine clinical practice, treatment interruption or inconsistent medication use may compromise therapeutic exposure and contribute to relapse, hospitalization, and functional deterioration [5,6,7].

Oral administration remains the most accessible and flexible strategy for psychiatric pharmacotherapy. However, its dependence on repeated patient-initiated dosing represents an important limitation in long-term treatment [8,9]. Difficulties with adherence may arise from multiple factors, including the chronic nature of psychiatric illness, adverse effects, impaired insight, cognitive dysfunction, complex treatment regimens, and individual preferences regarding medication [10,11,12]. Consequently, pharmaceutical strategies capable of maintaining therapeutic drug exposure while reducing the frequency of administration have become an important component of modern psychiatric pharmacotherapy [9,13]. Long-acting injectable (LAI) antipsychotics were developed to address some of these limitations by providing prolonged drug exposure after a single administration [14,15]. Current LAI formulations include oily solutions or suspensions, aqueous crystalline or nanosuspension systems, and biodegradable polymer-based depots [16,17,18]. Their use is supported by clinical guidelines, which consider LAIs an alternative to oral antipsychotics for adults with psychotic disorders requiring long-term treatment, with the choice guided by effectiveness, tolerability, patient preference, and treatment context [14].

Despite their established clinical utility and ability to provide sustained therapeutic exposure, currently available LAI formulations have distinct pharmaceutical and pharmacokinetic characteristics, with potential limitations that depend on both the active pharmaceutical ingredient and the formulation technology. Drug release from conventional depots is influenced by drug solubility, particle size, formulation composition, tissue environment, and the physicochemical properties of the carrier [19]. Poly(lactic-co-glycolic acid) (PLGA)-based systems, for example, can provide prolonged release through a combination of diffusion and polymer degradation, but their release profiles may include an initial burst followed by more complex phases of drug liberation. Polymer degradation may additionally modify the local microenvironment and influence drug stability and release kinetics [20,21,22,23]. These characteristics highlight the continuing need for delivery platforms capable of providing more predictable and tunable control of drug exposure.

Hydrogels have emerged as a versatile class of biomaterials with potential applications in controlled drug delivery [24,25,26,27,28,29]. They consist of three-dimensional polymeric networks capable of retaining substantial quantities of water, and their structural and physicochemical properties can be adjusted through polymer composition, crosslinking density, network architecture, degradation mechanisms, and drug–polymer interactions. Injectable and in situ-forming hydrogels are particularly relevant to the development of next-generation depot systems [30,31,32,33]. Such formulations can be administered as relatively low-viscosity materials and subsequently undergo gelation at the target site, generating a localized drug depot without requiring implantation of a preformed solid device [30,34]. The relevance of hydrogel-based delivery to psychiatric pharmacotherapy is increasingly supported by preclinical research. Reported outcomes include sustained in vitro drug release, prolonged systemic exposure, localized depot formation, and, in selected models, prolonged behavioral effects [31,35,36,37]. However, the available evidence remains heterogeneous, and most systems remain at the preclinical stage.

Although several reviews have addressed long-acting injectable antipsychotics or injectable hydrogel drug-delivery systems, these bodies of literature have largely evolved in parallel. Reviews of LAI antipsychotics have primarily focused on clinically available formulations, pharmacokinetic characteristics, administration strategies, and in vitro drug-release assessment, whereas broader reviews of injectable hydrogels have generally examined material design, gelation mechanisms, drug-loading strategies, and therapeutic applications across non-psychiatric fields [38,39,40,41]. To our knowledge, the intersection between hydrogel engineering and long-acting psychiatric pharmacotherapy has not been comprehensively examined from a translational perspective. Therefore, this narrative review addresses this gap and critically examines the current state of hydrogel-based depot systems in psychiatric pharmacotherapy, with particular emphasis on antipsychotic delivery. We summarize the limitations of existing long-acting delivery technologies, describe the physicochemical principles underlying hydrogel-based drug release, and evaluate preclinical evidence for hydrogel formulations of major antipsychotic agents. We further examine the pharmacokinetic, pharmacodynamic, behavioral, cognitive, and local-tolerability endpoints used in preclinical studies and discuss the principal barriers to clinical translation.

## 2. Method

This narrative review was conducted through a structured literature search aimed at identifying studies relevant to hydrogel-based drug-delivery systems with potential applications in psychiatric pharmacotherapy. PubMed, Scopus, Web of Science, Google Scholar, and ClinicalTrials.gov were searched for relevant publications from database inception to 2026. The search strategy combined terms related to hydrogel technologies, sustained and long-acting drug delivery, and psychiatric pharmacotherapy, including combinations of the following keywords: “hydrogel”, “injectable hydrogel”, “in situ gel”, “long-acting injectable”, “antipsychotic”, “psychiatric pharmacotherapy”, “risperidone”, “paliperidone”, “aripiprazole”, “olanzapine”, “quetiapine”, and “schizophrenia”. Boolean operators (AND/OR) were used to combine and refine the search terms.

Studies were considered eligible when they investigated hydrogel-based or in situ gel-assisted drug-delivery systems relevant to psychiatric pharmacotherapy, with each manuscript evaluated by two independent reviewers. Discrepancies were resolved through discussion and consensus. Both in vitro and in vivo studies were considered, together with clinically relevant evidence where available. Given the heterogeneous and predominantly preclinical nature of the field, no restrictions were imposed on hydrogel composition, crosslinking mechanism, or administration route. Publications were excluded when they did not involve a hydrogel or in situ gel-assisted delivery platform, addressed therapeutic areas unrelated to the scope of the review, provided insufficient information, or represented conference abstracts, editorials, or commentaries. Only articles published in English were considered. The reference lists of eligible original studies and pertinent review articles were additionally screened manually to identify studies not retrieved during the initial database search.

**Figure 1:**
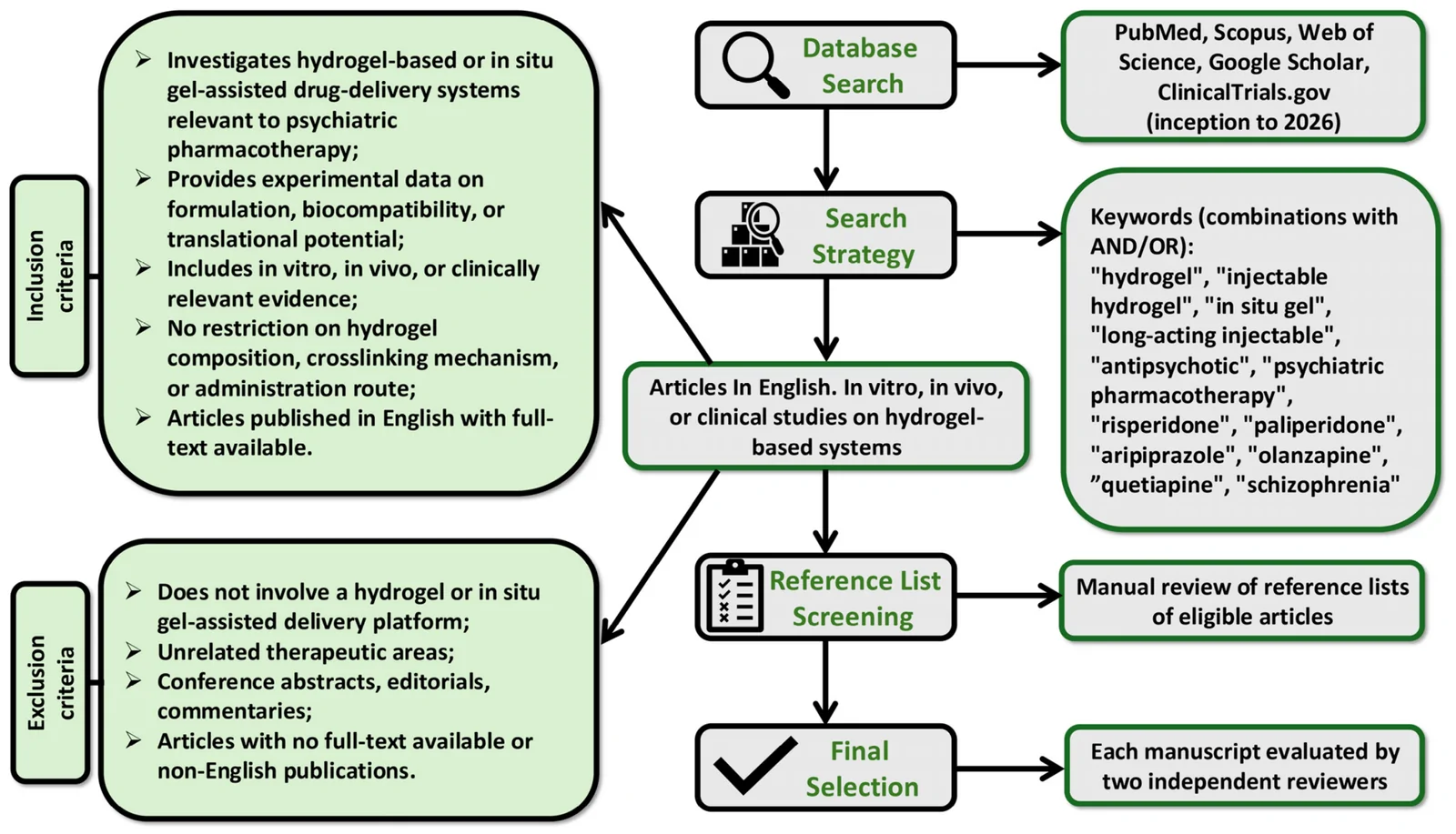
Literature search and study-selection process of hydrogel-based drug-delivery systems in psychiatric pharmacotherapy.

As a narrative rather than systematic review, the study was not conducted according to the Preferred Reporting Items for Systematic Reviews and Meta-Analyses (PRISMA) framework and no formal meta-analysis was performed. The overall literature-selection strategy is summarized in Figure 1.

## 3. Current Long-Acting Drug Delivery Strategies in Psychiatric Pharmacotherapy

### 3.1. Oral Therapy and Adherence Limitations

Oral administration remains the most widely used route for psychiatric pharmacotherapy because it is non-invasive, flexible, relatively inexpensive, and readily reversible [42]. However, its principal limitation in chronic psychiatric treatment is the requirement for repeated patient-initiated administration. Daily dosing creates multiple opportunities for delayed or missed doses, particularly in disorders characterized by impaired insight, cognitive dysfunction, disorganization, adverse effects, substance use, or fluctuating motivation [43,44]. Medication adherence is therefore not simply a behavioral issue but an important determinant of pharmacokinetic exposure [45].

Missing individual doses can produce reductions in plasma drug concentrations, while repeated or prolonged non-adherence may result in loss of therapeutic exposure and an increased probability of clinical deterioration [45,46]. Conversely, repeated administration of an oral drug can generate fluctuations between peak and trough concentrations, depending on the drug’s absorption characteristics, half-life, dosing interval, and formulation [47,48].

### 3.2. Conventional LAI Formulations

LAIs attempt to address several of oral medication limitations by reducing the number of administration events and making medication administration directly observable [38]. However, evidence comparing LAIs with oral antipsychotics is heterogeneous and strongly influenced by study design [49,50]. A large systematic review and meta-analysis found that observational and pre–post studies generally favored LAIs for outcomes such as hospitalization or relapse, whereas randomized controlled trials have produced less consistent differences [49]. Thus, the rationale for long-acting delivery should not be reduced to the assumption that injectable formulations are universally more efficacious than oral formulations. Rather, the principal pharmacotherapeutic rationale is the possibility of maintaining treatment continuity and providing more predictable drug exposure while reducing the burden of frequent administration.

Conventional LAI antipsychotics employ several formulation strategies. Oily depots rely primarily on slow dissolution and diffusion of the active compound from the injection site. The physicochemical properties of the drug and vehicle determine depot persistence and systemic absorption [51]. Representative oil-based LAI antipsychotics include haloperidol decanoate [52,53] and fluphenazine decanoate [54,55], which are formulated as decanoate esters in sesame oil, as well as flupentixol decanoate and zuclopenthixol decanoate, which are formulated as decanoate esters in a vegetable-oil vehicle [55,56] (Table 1).

Other LAI antipsychotics are formulated as aqueous suspensions or nanosuspensions, including olanzapine pamoate, paliperidone palmitate, and aripiprazole monohydrate [38,55]. Although these formulations can provide prolonged exposure, injection-site discomfort and local reactions remain relevant considerations [18].

### 3.3. PLGA-Based Depot Systems

PLGA has become one of the most widely investigated biodegradable polymers for controlled parenteral drug delivery [22,57]. The polymer consists of lactic acid and glycolic acid units, and its degradation occurs predominantly through hydrolysis of ester bonds. Water penetration into the polymer matrix initiates hydrolysis, progressively altering polymer molecular weight, hydrophobicity, porosity, and drug-release characteristics [58]. Polymeric microspheres provide a different mechanism of sustained delivery. The drug is incorporated within biodegradable polymeric particles, most commonly PLGA, from which the active compound is released over time through a combination of diffusion and polymer degradation. Risperidone was developed as a long-acting injectable formulation based on polymeric microspheres [59]. Risperidone is microencapsulated within biodegradable PLGA microspheres, which are administered as an aqueous suspension after reconstitution. The microspheres gradually release risperidone through polymer degradation and drug diffusion, providing sustained drug exposure after intramuscular administration [38]. The release characteristics can be modulated by the properties of the PLGA polymer, including its molecular weight and lactic acid-to-glycolic acid ratio, as well as by microsphere size and size distribution [55].

## 4. Hydrogels as Drug Delivery Platforms

### 4.1. Definition and Physicochemical Characteristics

Hydrogels are three-dimensional (3D) polymeric networks capable of retaining substantial amounts of water while maintaining an insoluble or semi-solid network structure [32]. Their physicochemical properties can be modified through polymer selection, crosslinking density, network architecture, degradation mechanisms, and incorporation of additional drug carriers [60]. Unlike conventional solid polymeric microspheres, hydrogels provide a highly hydrated environment in which drug transport can occur through diffusion, swelling, polymer relaxation, degradation, or combinations of these mechanisms. This creates a broad design space for controlling drug release [40,61,62].

Hydrogel-based systems are not inherently exempt from an initial burst release, the magnitude of which may vary according to formulation-specific factors. Although burst release may occur in both PLGA-based depots and hydrogel systems, the underlying mechanisms are formulation-dependent. In PLGA systems, early drug release may result from rapid diffusion of drug located near the polymer surface before degradation-mediated release becomes predominant [22,63], whereas in hydrogels it may arise from rapid diffusion of loosely associated or surface-localized drug through the hydrated polymer network, with its magnitude influenced by water content, porosity, mesh size, crosslinking density, swelling behavior, mechanical strength and drug–polymer interactions [64,65,66]. Water content influences drug solubilization and diffusion [67,68]. Increasing swelling generally increases the aqueous phase available for drug transport, although the relationship between swelling and release is not necessarily linear because increased swelling may also modify polymer–drug interactions and network dimensions [69,70].

Mesh size represents the effective spacing within the polymer network through which molecules can diffuse. It is determined by polymer concentration, molecular weight, crosslinking density, and the physicochemical characteristics of the network. A smaller mesh size can retard the movement of larger molecules, whereas a larger network may facilitate faster diffusion [61,71,72]. Crosslinking can be achieved through physical or chemical mechanisms. Hydrogel network formation can be classified according to the predominant crosslinking or gelation mechanism as physical, chemical/covalent, ionic, enzymatic, or stimuli-responsive. Physical crosslinking relies on reversible non-covalent interactions, including hydrogen bonding, hydrophobic associations, crystallization, and chain entanglement, whereas chemical crosslinking involves the formation of covalent bonds and generally provides greater structural stability [73,74,75,76]. Ionic crosslinking results from electrostatic interactions between charged polymer chains and multivalent ions, while enzymatic crosslinking uses enzyme-mediated reactions to generate the polymer network under comparatively mild conditions [77,78]. Stimuli-responsive systems undergo gelation or changes in network structure in response to environmental triggers such as temperature, pH, or ionic strength, making them particularly relevant for in situ-forming drug depots [77,79]. These categories may overlap, as a single formulation can combine more than one crosslinking or gelation mechanism. The selected strategy influences mechanical stability, degradation, injectability, drug release, and biocompatibility and should therefore be matched to the intended administration route and therapeutic application.

### 4.2. Hydrogel-Based Antipsychotic Delivery

For long-acting psychiatric drug delivery, injectability is particularly important. The formulation must be sufficiently fluid to pass through an appropriate needle or administration device while maintaining adequate depot integrity after administration [80,81]. Recent injectable hydrogel research has emphasized shear-thinning behavior, rapid self-healing, controlled gelation, and localized drug release as key formulation characteristics [40,76,82].

The available literature indicates that hydrogel-based antipsychotic delivery remains predominantly preclinical [31,37,82,83,84]. Importantly, the investigated systems are not uniform: some are injectable depots [82], or transdermal [84,85], whereas others are intranasal in situ-forming gels or hydrogel-forming microneedle systems [31]. Therefore, their pharmacokinetic objectives and clinical implications differ. The current evidence concerns risperidone (RIS), aripiprazole (APZ), olanzapine (OLZ), paliperidone (PLPD), paliperidone palmitate (PPP), quetiapine (QTP) and the investigational antipsychotic peptide PAOPA (Table 2).

The suitability of a hydrogel architecture for a given antipsychotic depends not only on the properties of the polymer network, but also on drug-specific characteristics, including molecular weight, lipophilicity, ionization state, aqueous solubility, affinity for the polymer matrix, and intended drug loading. These factors determine whether release is governed predominantly by diffusion, swelling, drug–polymer interactions, or matrix degradation [86,87,88]. Drug loading represents an additional formulation constraint for highly hydrophobic antipsychotics. Because conventional hydrogels are predominantly aqueous and hydrophilic, limited drug solubility may restrict the amount of active compound that can be incorporated homogeneously into the network. Drug partitioning between the aqueous phase, polymer matrix, and any incorporated hydrophobic carrier can further determine loading efficiency and subsequent release behavior. At high drug concentrations, insufficient compatibility with the hydrogel matrix may promote precipitation, crystallization, or aggregation, potentially producing heterogeneous drug distribution and altering both early and sustained release. Accordingly, poorly water-soluble antipsychotics may benefit from architectures incorporating solubilizing or lipid-based carriers, such as cyclodextrins, polymeric micelles, nanotransfersomes, lipid-core nanocapsules, or lipospheres, whereas injectable cross-linked or thermoresponsive networks may be more appropriate when localized depot formation and prolonged release are the principal objectives [89,90]. The formulations summarized in Table 2 therefore represent drug-specific combinations of active pharmaceutical ingredient, carrier architecture, and administration route rather than interchangeable hydrogel platforms.

**Table 2:** Hydrogel-based delivery systems for antipsychotic agents.

Antipsychotic	Hydrogel/Delivery System	Route	Experimental Model	Key Limitation	Ref.
Risperidone	Carbomer 934 transdermal hydrogel with chemical penetration enhancers	Transdermal	In vitro: Porcine skin was peeled from suckling Yorkshire pigs. In vivo: Rabbits	Very small sample (n = 3). No chronic-dose evaluation. No long-term skin tolerability reported—delivery evaluated only up to 48 h.	[85]
Risperidone	PVA/PVP hydrogel-forming microneedle array patches (HFMAPs) combined with a RIS–HP-β-CD physical-mixture tablet reservoir	Transdermal	Female Sprague–Dawley rats	Healthy rats. No schizophrenia model or behavioral assessment.	[84]
Risperidone	Thermoresponsive poly(N-acryloyl glycinamide) (PNAGA124) hydrogel, 6% *w*/*v*; formulation additionally evaluated with Mg(OH)_2_	Intended for local administration by subcutaneous injection of a warm solution followed by gelation	In vitro only	No in vivo/animal experiments.	[91]
Risperidone	Silk fibroin hydrogel; methanol- or HCl/acetone-induced gelation	Proposed injectable implant; route not evaluated in vivo	In vitro only	No in vivo/animal experiments.	[30]
Risperidone	PLGA (75:25)-based in situ gel-forming implant (ISGFI) using DMSO as solvent	Subcutaneous injection	Adult male albino rats; healthy rats for pharmacokinetics and ketamine-induced schizophrenia-like rats	Small groups (n = 5).	[92]
Risperidone	Thermosensitive PLGA–PEG–PLGA ISFG; optimized formulation 50% *w*/*w* PLGA–PEG–PLGA in NMP; compared with PLGA 33% *w*/*w* in situ-forming implant (ISFI)	Subcutaneous injection	Healthy male New Zealand rabbits	Very small sample (n = 3/group); healthy rabbits rather than a disease model. No behavioral/cognitive assessment.	[82]
Risperidone	Thermoresponsive chitosan/β-glycerol phosphate (βGP) hydrogel using non-conventional β-chitosan derived from squid pen or cuttlebone	Injectable in situ-gelling formulation, preformed hydrogel and xerogel	In vitro only	No in vivo/animal experiments.	[36]
Risperidone	Ion-sensitive gellan gum-based in situ hydrogel combined with cellulose derivatives and containing risperidone-loaded Pluronic F108/F127 polymeric micelles	Intranasal (intended)	In vitro only	No in vivo/animal experiments.	[93]
Risperidone	Carbomer-based hydrogel containing risperidone-loaded solid lipid nanoparticles	Oral transmucosal (intended)	In vitro only	No in vivo/animal experiments.	[94]
Risperidone	Thermo-responsive UCST hydrogels based on poly(N-acryloyl glycinamide) (PNAGA) and poly(NAGA-co-NALALA) copolymers	Potential injectable depot	In vitro only	No in vivo/animal experiments.	[95]
Risperidone	Hydrogel-template-fabricated homogeneous risperidone-loaded PLGA microcylinders	Not administered; in vitro drug-release system	In vitro only	Hydrogel serves only as a fabrication template, it is not the final drug depot. No in vivo administration.	[96]
Paliperidone	In situ gel based on Carbopol 934 + HPMC K4M, with or without HP-β-cyclodextrin (HP-β-CD) inclusion complex	Intranasal	In vitro: dialysis membrane. Ex vivo: freshly excised sheep nasal mucosa	No in vivo/animal experiments.	[97]
Paliperidone palmitate	Injectable alginate in situ-forming hydrogel	Intended intramuscular injection	In vitro only. Additionally evaluated in human plasma.	No in vivo/animal experiments.	[98]
Aripiprazole	Molecularly imprinted pHEMA cryogel patch	Proposed transdermal delivery	In vitro drug-release study. Cytotoxicity in L929 mouse fibroblasts	No animal/in vivo study. No actual skin permeation experiment.	[37]
Aripiprazole	Ion-sensitive in situ gellan gum containing APZ-loaded nanotransfersomes (APZ-TFS-Gel)	Intranasal	In vitro + in vivo, male NMRI albino mice; ketamine-induced psychosis model	Short-term pharmacodynamic model. No chronic dosing/safety assessment. Sustained release demonstrated only to 48 h.	[99]
Aripiprazole	Injectable in situ-forming alginate hydrogel	Injected into the muscular tissue of pig necks	Mainly in vitro/ex vivo formulation study	No in vivo administration. Depot formation was demonstrated in excised pig muscle, not in a live pig. Therefore, long-acting efficacy remains formulation proof-of-concept.	[100]
Aripiprazole	Agar/β-cyclodextrin composite cryogel	Potential peroral delivery	In vitro only	No in vivo/animal experiments.	[101]
Olanzapine	Nanoparticle network hydrogel (NNH) consisting of hydrophobically modified starch nanoparticles (SNPs) and mucoadhesive chitosan oligosaccharide lactate (COL)	Intranasal	Male Sprague–Dawley rats. Amphetamine-induced model of schizophrenia-like symptoms	Lack of chronic repeated-dose toxicity data and uncertainty regarding the predominant drug-delivery mechanism.	[31]
Olanzapine	Thermosensitive Poloxamer 407 (P-407) hydrogel containing olanzapine-loaded lipid-core nanocapsules (HG-LCN)	Intended for intramuscular injection	In vitro only	No in vivo/animal experiments.	[102]
Olanzapine	OLZ/RDPA in situ thermoresponsive hydrogel	Intranasal	In vitro: Mouse brain microvascular endothelial (bEnd.3) cells and mouse hippocampal neuronal cell line (HT22 cells) In vivo: male Wistar rats; CUMS depression model induced for 4 weeks	The therapeutic strategy was evaluated specifically in a CUMS depression model rather than in an antipsychotic indication such as schizophrenia.	[103]
Olanzapine	Hydrogel-forming microarray patch (MAP) consisting of cross-linked PVA/PVP microneedle arrays	Transdermal	In vitro: neonatal porcine skin. In vivo: healthy female Sprague–Dawley rats	Healthy rats, without evaluation in a psychiatric disease model, long-term/repeated-dose studies, or behavioral efficacy assessment.	[104]
Olanzapine	Hydrogel-forming microneedle (MN) arrays combined with a liquid drug reservoir containing OLZ	Transdermal	In vitro: neonatal porcine skin	No in vivo/animal experiments.	[105]
Quetiapine fumarate	Bio-shielding in situ-forming gel (BSIFG) loaded with quetiapine fumarate (QTP-F) lipospheres	Intramuscular depot injection	In vitro: formulation characterization, injectability and QTP-F release In vivo: male New Zealand albino rabbits	No behavioral efficacy assessment, psychiatric disease model, or repeated-dose evaluation.	[106]
Quetiapine fumarate	pH-sensitive Gel/HPMC hybrid hydrogel, prepared by graft polymerization of gelatin (Gel) and hydroxypropyl methylcellulose (HPMC) and chemically cross-linked with glutaraldehyde (GA)	Oral	In vitro: formulations; swelling (dynamic/equilibrium), diffusion, sol–gel fraction, porosity, drug content and QTP-F release In vivo: Albino rabbits, male and female	No psychiatric disease model, behavioral efficacy assessment or chronic/repeated-dose evaluation. Moreover, this is an oral controlled-release hydrogel rather than an injectable/localized depot, limiting its direct relevance to LAI hydrogel systems.	[107]
Quetiapine hemifumarate	Cross-linked hyaluronic acid (cHA)-based hydrogel formulation	Subcutaneous injection	In vitro: hydrogel swelling/drug loading optimization; inversion test; rheology; shear-thinning and injectability; QTP release; release-kinetic modeling In vivo degradation/toxicity: male ICR mice, SC injection; depot degradation; serum chemistry and hematoxylin and eosin (H&E) histology In vivo pharmacokinetic: male Sprague–Dawley rats	No schizophrenia/psychosis model or behavioral efficacy assessment.	[108]
Quetiapine fumarate	HPMC K15M-based hydrophilic sustained-release matrix tablet forming a hydrated gel layer in aqueous media	Oral sustained-release tablets	In vitro only	No in vivo/animal experiments. The delivery system is an oral hydrophilic matrix tablet that forms a gel layer after hydration, rather than an injectable/localized hydrogel depot, so its relevance to long-acting depot systems is limited	[109]
PAOPA	In situ-gelling starch nanoparticle (SNP)/O-carboxymethyl chitosan (CMCh) nanoparticle network hydrogel	Intranasal	In vitro: gelation/degradation, mechanics, mucoadhesion, cytocompatibility and PAOPA release/stability. In vivo: Male Sprague–Dawley rats; MK-801-induced schizophrenia-like model	PAOPA remains an experimental antipsychotic candidate, and the study did not include a conventional antipsychotic comparator.	[83]

## 5. Preclinical Models for Evaluating Hydrogel-Based Antipsychotic Delivery

The preclinical evaluation of hydrogel-based antipsychotic delivery systems requires a broader experimental framework than the demonstration of sustained drug release alone. Evaluation of a hydrogel-based long-acting formulation must simultaneously characterize the active pharmaceutical ingredient, the carrier, the drug–material interaction, the administration procedure, depot formation and degradation, and the resulting pharmacokinetic–pharmacodynamic relationship. These variables are particularly important for psychiatric therapeutics because prolonged drug exposure is clinically meaningful only if adequate central pharmacological activity is maintained without excessive initial exposure, cumulative toxicity, or unacceptable local reactions [40,86,110,111].

A rational preclinical programme should therefore integrate pharmacokinetic studies in healthy animals, pharmacodynamic evaluation in disease-relevant experimental models, behavioral testing across complementary schizophrenia-related domains, local-tolerability assessment, and, where appropriate, larger-animal studies performed at clinically relevant injection volumes [112,113,114,115]. Importantly, no experimental animal reproduces schizophrenia as a whole [116]. Rodent models instead reproduce selected neurobiological mechanisms or measurable endophenotypes associated with the disorder, such as dopaminergic hyperresponsivity, the hypofunction of the N-methyl-D-aspartate (NMDA) receptor, impaired sensorimotor gating, social withdrawal, or cognitive dysfunction [117,118,119,120,121,122]. Accordingly, a delivery platform intended for antipsychotic therapy should ideally be evaluated using more than one experimental endpoint and, at advanced stages, more than one model.

### 5.1. Rodent Models of Psychosis/Schizophrenia

Rodents are commonly used for initial pharmacokinetic screening because they permit serial comparison of formulations with relatively low material requirements [123,124,125,126,127,128].

For hydrogel-based antipsychotic development, model selection should follow the intended scientific question rather than attempting to identify the best schizophrenia model (Table 3). Acute amphetamine/methamphetamine [121,129,130,131], or ketamine paradigms [121,132,133,134,135,136] are appropriate for early proof-of-concept when the primary aim is to determine whether prolonged drug exposure extends a measurable antipsychotic-like effect. Conversely, prenatal methylazoxymethanol acetate (MAM) model [121,137,138,139], neonatal ventral hippocampal lesion (NVHL) model [121,140,141,142,143], maternal immune activation (MIA) model, or genetic models [121,144,145,146,147,148,149] become increasingly valuable when the formulation is sufficiently mature to justify evaluation of chronic treatment and repeated-dose efficacy.

### 5.2. Behavioral and Functional Assessment

Behavioral testing is essential for connecting sustained exposure to sustained pharmacological activity. The key methodological problem is that no rodent behavior is equivalent to hallucinations, delusions, avolition, or formal thought disorder [170,171]. Instead, experimental tasks quantify operationally defined constructs that can be related, with varying degrees of translational validity, to positive, negative, cognitive, or information-processing abnormalities observed in schizophrenia (Table 4).

### 5.3. Large-Animal Models

Rodent models remain essential for early formulation screening, pharmacokinetic characterization, and proof-of-concept studies. However, they do not fully reproduce the anatomical and physiological conditions encountered with human intramuscular or subcutaneous depots. The ratio between injection volume and local tissue mass differs substantially from that in humans, as do tissue architecture, vascularization, mechanical forces, depot geometry, and local clearance. Consequently, larger-animal models become increasingly relevant as a formulation progresses toward clinical translation [183,184].

Rabbits have been used extensively for the pharmacokinetic evaluation of long-acting formulations, including risperidone, paliperidone palmitate and quetiapine systems. Their larger body size permits administration of larger volumes and serial blood sampling, although rabbit muscle anatomy still differs substantially from that of humans [82,107,185,186].

Pigs are particularly attractive for injection-site studies because the dimensions and organization of porcine skin, subcutaneous tissue, and skeletal muscle allow administration using devices and volumes closer to those intended clinically. Ex vivo pig muscle has already been used to investigate depot formation of an injectable alginate–aripiprazole formulation [100]. However, ex vivo tissue cannot reproduce perfusion, inflammation, immune response, degradation, or systemic pharmacokinetics; living-animal studies are required to evaluate these phenomena.

At a different level of translation, non-human primates (NHPs) offer particular value for assessing pharmacodynamic and cognitive outcomes relevant to psychiatric disorders. Their role is therefore complementary to that of rabbits or pigs; rather than simply providing a larger injection site, NHPs can bridge part of the neurobiological gap between rodent behavioral models and human psychiatric disease. This is especially relevant to schizophrenia, in which higher-order cognitive dysfunction involves prefrontal cortical systems that are not fully reproduced in rodents. Indeed, the lateral prefrontal cortex has distinctive organization in anthropoid primates, making some aspects of working-memory circuitry difficult to investigate in conventional rodent models [187].

Pharmacological NHP models based on N-methyl-D-aspartate receptor hypofunction illustrate this translational potential. In adult marmosets (*Callithrix penicillata*), acute systemic MK-801 impaired spontaneous object recognition, eliminating the normal preference for a novel over a familiar object after a 6-h retention interval. The authors used the spontaneous object recognition paradigm as a measure of visual recognition memory and noted the increasing use of marmosets as translational models for neuropathological conditions. Importantly, NMDA-receptor antagonism produces recognition-memory impairment across species: the same paper discusses detrimental effects of NMDA-receptor antagonists in rodents, marmosets, rhesus monkeys, and human volunteers, supporting cross-species translation of this pharmacological approach [188]. A complementary model has been demonstrated in rhesus macaques (*Macaca mulatta*) exposed to subanesthetic ketamine. Roussy et al. trained rhesus monkeys to perform a naturalistic visuospatial working-memory task in a virtual environment while recording neuronal activity from the lateral prefrontal cortex. Ketamine produced transient working-memory impairment while largely sparing perceptual and motor performance and was associated with disruption of prefrontal neuronal coding of remembered spatial information. The paradigm therefore provides access not only to behavioral endpoints but also to neural mechanisms directly relevant to the cognitive dysfunction associated with schizophrenia. In particular, ketamine significantly reduced working-memory performance shortly after administration, whereas performance in the perception-control condition remained unaffected, supporting a relatively selective cognitive effect [187].

The value of a large-animal study therefore lies not simply in increasing animal size, but in reproducing the intended human administration conditions as closely as possible. Needle gauge, injection speed, injected volume, anatomical site, formulation temperature, and handling procedure should approximate the intended clinical product whenever feasible.

## 6. Safety, Biocompatibility and Injection-Site Considerations

The potential clinical advantages of hydrogel-based long-acting antipsychotic delivery depend not only on sustained drug release but also on the biological response generated by the formulation during its residence at the administration site. Injectable hydrogels and in situ-forming depots inevitably interact with extracellular matrix components, resident immune cells, vasculature, and surrounding connective or muscular tissue. Following implantation or injection of a biomaterial, the local response may include protein adsorption, acute and chronic inflammation, macrophage recruitment, foreign-body giant-cell formation, and, in some cases, fibrous encapsulation [189]. The magnitude and duration of this response depend on several formulation-related properties, including polymer chemistry, surface characteristics, depot dimensions, degradation kinetics, residual solvents, crosslinking agents, and the mechanical properties of the material. Consequently, apparent biocompatibility in short-term cell-viability assays should not be considered sufficient evidence of long-term local tolerability [190,191].

Long-term assessment of inflammatory-cell infiltration, fibrosis, necrosis, edema, local vascular changes, and tissue recovery after complete depot degradation therefore remains necessary before repeated administration can be considered safe. This is particularly important for psychiatric treatments, in which administration may continue for years and successive injections may repeatedly expose the same or adjacent anatomical regions to biomaterials. Changes in the local microenvironment can influence drug stability and release kinetics, while rapid accumulation of degradation products may generate transient acidic conditions within the polymer matrix [188].

Other hydrogel systems, including those based on chitosan, alginate, hyaluronic acid, gelatin, starch derivatives, or synthetic thermoresponsive polymers, have different degradation pathways and therefore require material-specific characterization. The relevant question is not merely whether a polymer is biodegradable, but whether the intact material, degradation intermediates, residual crosslinkers, solvents, and final degradation products remain locally and systemically tolerated throughout the entire residence period [192,193,194].

A major rationale for depot development is the possibility of reducing administration frequency. In principle, a hydrogel that maintains therapeutically relevant exposure for weeks rather than hours or days could reduce the number of treatment events required and thereby lessen dependence on repeated patient-initiated dosing [195,196]. This concept is supported at the preclinical level by several systems included in the present review. However, reduced dosing frequency should be regarded as a potential clinical consequence of an appropriate pharmacokinetic profile rather than an intrinsic property of hydrogels. The relevant translational endpoint is sustained exposure within an effective and tolerable concentration range, not simply prolonged drug detectability.

Localized depot formation provides another important opportunity. In situ-gelling formulations can be administered in a relatively fluid state and subsequently form a semisolid or solid-like reservoir at the site of administration, potentially limiting immediate drug dispersion and providing a defined compartment from which release can be controlled [197,198]. Long-acting formulations can also reduce peak-to-trough fluctuations relative to some oral dosing regimens, and pharmacokinetic characteristics such as absorption rate and peak concentration may affect adverse-effect burden [47]. Psychiatric LAIs also have pharmacokinetic requirements that extend beyond maintaining therapeutic exposure and limiting excessive peak concentrations. With repeated administration, drug accumulation and the time required to reach steady state must be characterized, particularly because depot absorption may become the rate-limiting step and can generate prolonged apparent or terminal half-lives. These properties determine both the extent of accumulation during maintenance treatment and the persistence of systemic exposure after treatment discontinuation. Interindividual variability is also clinically relevant, as absorption from the injection site, formulation characteristics, administration technique, injection site, body composition, metabolic factors, and concomitant medications may influence systemic exposure. Furthermore, the pharmacokinetic consequences of delayed or missed injections should be established for each formulation. A sufficiently prolonged release profile may provide some protection against short treatment gaps, but longer delays may allow concentrations to fall below the therapeutic range and may require formulation-specific reinitiation or loading strategies.

Clinically relevant injectability should also be characterized through parameters such as syringeability, injection force, needle gauge, injection rate, and formulation temperature. Gelation time must be sufficiently short to ensure reliable depot formation after administration, while remaining long enough to permit practical handling and injection. The resulting gel should subsequently maintain adequate residence time and structural integrity at the administration site to support the intended release period, without compromising local tolerability or biodegradation. These parameters are interdependent and should therefore be optimized together rather than evaluated in isolation [199,200].

Sterilization represents a particularly important translational challenge for injectable hydrogel formulations because the sterilization procedure must ensure microbial safety without compromising polymer structure, gelation behavior, drug stability, or subsequent release kinetics. Sterile filtration may be suitable for sufficiently low-viscosity precursor solutions and avoids direct exposure to heat or ionizing radiation, but it becomes impractical for highly viscous formulations, particulate systems, or preformed hydrogels [201,202].

Local tolerability is also influenced by administration- and formulation-specific parameters beyond polymer biocompatibility. Formulation pH and osmolality should remain within ranges compatible with the intended tissue environment, as substantial deviations may contribute to local irritation, pain, cellular injury, or inflammatory responses. Injection volume is similarly relevant, because larger depot volumes may increase local tissue distension and mechanical pressure and may influence both patient discomfort and the extent of the local tissue response [203,204]. These factors should be considered together with polymer composition, crosslinking chemistry, degradation behavior, and depot persistence when evaluating the injection-site safety of a long-acting hydrogel formulation.

An optimal system should produce reproducible localized depot formation, controlled early and maintenance release, predictable degradation, minimal persistent inflammatory response, and systemic exposure sufficient for efficacy without unnecessary peak concentrations. Reduced administration frequency and the possibility of avoiding oral supplementation are potentially important clinical advantages, but they must be balanced against injection-site effects and the limited reversibility inherent to long-acting treatment.

## 7. From Preclinical Proof-of-Concept to Clinical Translation

Translation of hydrogel-based antipsychotic delivery systems into clinically applicable therapies requires progression beyond formulation development and proof-of-concept toward increasingly comprehensive in vivo evaluation of pharmacokinetics, therapeutic efficacy, safety, and tolerability.

As summarized in Table 2, the available evidence on hydrogel-based antipsychotic delivery is heterogeneous in terms of formulation design, administration route, and level of preclinical evaluation. Risperidone accounts for the largest number of investigated systems, encompassing transdermal hydrogels and hydrogel-forming microneedle patches [84,85], thermoresponsive and injectable in situ-forming formulations [36,82,91,92,95], and intranasal, oral transmucosal, and hydrogel-assisted delivery approaches [30,93,94,96]. Nevertheless, much of this evidence remains limited to in vitro evaluation [30,36,91,93,94,95,96], while the in vivo studies were conducted in small groups or predominantly in healthy animals [82,84,85,92].

The evidence for paliperidone and paliperidone palmitate is more limited, comprising an intranasal in situ gel evaluated in vitro and ex vivo [97] and an injectable alginate hydrogel investigated in vitro and in human plasma [98], respectively, without in vivo animal evaluation.

Aripiprazole has been explored through transdermal, intranasal, injectable, and potential oral hydrogel-based approaches [37,99,100,101]. Among these, the intranasal gellan gum formulation was evaluated in vivo in a ketamine-induced psychosis model in mice [99], whereas the remaining systems were restricted to in vitro or ex vivo assessment [37,100,101]. A similarly diverse range of delivery routes has been investigated for olanzapine, including intranasal [31,103], intramuscular [102], and transdermal systems [104,105]. In vivo evaluation included an amphetamine-induced schizophrenia-like model [31], a CUMS depression model [103], and healthy rats [104], whereas other formulations remained at the in vitro stage [102,105].

Quetiapine-based systems have included intramuscular [106], subcutaneous [108], and oral hydrogel or gel-forming formulations [107,109], with in vivo studies conducted in rabbits, mice, or rats but without psychiatric disease models or behavioral efficacy assessment [106,107,108,109]. Finally, PAOPA was investigated using an intranasal in situ-gelling nanoparticle network hydrogel in an MK-801-induced schizophrenia-like rat model [83]. Unlike the approved antipsychotics summarized above, PAOPA remains an experimental antipsychotic candidate, and the study did not include a conventional antipsychotic comparator.

A substantial proportion of the investigated systems have not progressed beyond in vitro or ex vivo characterization, while in vivo studies frequently rely on small experimental groups, healthy animals, or short-term pharmacodynamic models. Even among formulations evaluated in disease-related models, chronic or repeated-dose assessment and longer-term safety evaluation are generally limited. These differences also make direct comparison between formulations difficult, particularly because the investigated systems span markedly different routes of administration and therapeutic objectives, ranging from injectable depots to intranasal and transdermal delivery.

Accordingly, progression toward clinical translation will require a more sequential and standardized preclinical evaluation strategy. Formulations showing favorable physicochemical characteristics and sustained drug release in vitro should subsequently be assessed for injectability or route-specific administration performance, stability, and reproducibility before advancing to pharmacokinetic studies in vivo. Pharmacokinetic characterization should establish whether the intended prolonged exposure is achieved under physiological conditions, while pharmacodynamic and behavioral studies in relevant experimental models are needed when therapeutic efficacy is an objective of the delivery system. Systemic toxicity, depot degradation, and repeated-dose effects also require evaluation before progression toward larger-animal studies and formal toxicological assessment. Importantly, the translational value of emerging hydrogel-based systems should be assessed against established LAI formulations that already provide effective sustained drug exposure, recognizing that their pharmacokinetic characteristics, administration requirements, and clinical limitations are specific to the active pharmaceutical ingredient and formulation technology.

An additional translational consideration for stimuli-responsive hydrogels is the predictability of the physiological trigger under in vivo conditions. Although temperature-, pH-, ion-, enzyme-, and redox-responsive systems provide attractive mechanisms for controlling gelation or drug release, the corresponding physiological stimuli may vary between individuals, anatomical sites, and local tissue conditions. Local inflammation, tissue perfusion, metabolic activity, ionic composition, enzymatic activity, or changes in the microenvironment surrounding the depot may further alter the magnitude or duration of the stimulus [205,206,207,208,209]. Consequently, a responsive formulation that performs reproducibly under tightly controlled in vitro conditions may exhibit greater variability in gelation, degradation, or drug-release kinetics in vivo. For long-term depot applications, the relevant physiological trigger should therefore operate within a sufficiently predictable range, and formulation performance should be evaluated across physiologically relevant variations in that stimulus before clinical translation.

For degradable hydrogels, the polymer network is not static during the intended release period. Progressive cleavage of crosslinks or polymer chains can reduce network density, increase effective mesh size and porosity, and alter swelling and mechanical integrity, thereby modifying the relative contributions of diffusion and degradation to drug release. Consequently, a formulation that initially exhibits diffusion-controlled release may become increasingly degradation- or erosion-dependent as the network evolves. This is particularly relevant for long-acting depots intended to function over weeks or months, because non-linear degradation, local microenvironmental changes, or accelerated structural loss may produce changes in release rate and potentially less predictable systemic exposure.

Reduced administration frequency may represent a clinically relevant advantage of appropriately designed hydrogel depots. However, avoidance of oral supplementation during treatment initiation cannot be assumed from sustained release alone. This would require demonstration that the formulation provides sufficiently rapid early drug release to achieve therapeutic systemic concentrations soon after administration while subsequently maintaining exposure within the therapeutic range. For hydrogel systems with delayed early release or a lag phase, an oral overlap or alternative loading strategy may still be necessary. Current preclinical evidence is insufficient to establish that the investigated hydrogel formulations consistently eliminate this requirement.

**Figure 2:**
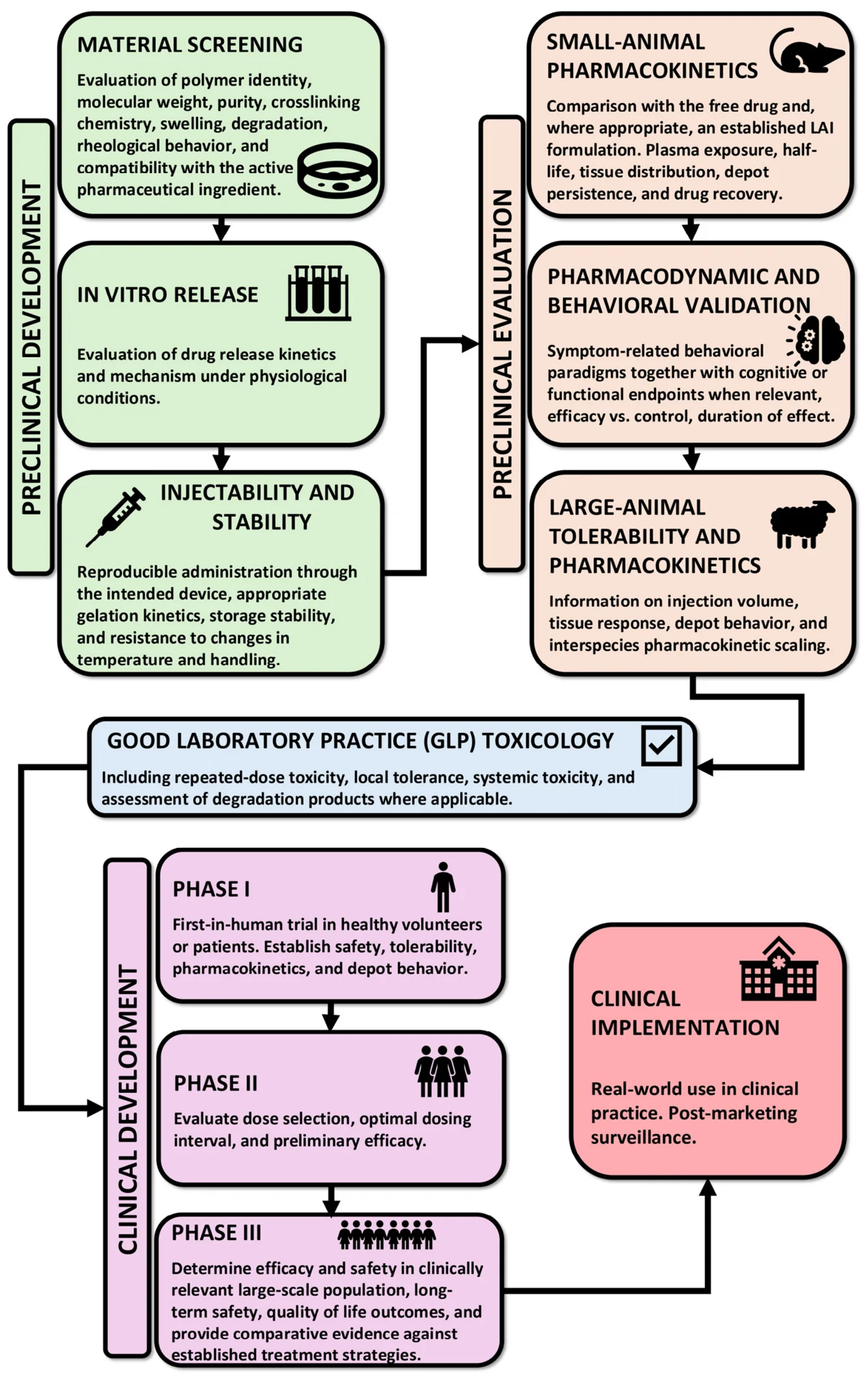
Proposed translational roadmap for hydrogel-based long-acting antipsychotic delivery systems, from material and formulation development through preclinical pharmacokinetic, pharmacodynamic, behavioral, and toxicological evaluation to clinical development and implementation. The proposed sequence integrates pharmaceutical-development considerations with established principles for nonclinical safety evaluation and progression to human clinical trials.

Ultimately, successful translation will depend not only on demonstrating prolonged drug release, but on establishing that this release can be achieved reproducibly and safely while providing a clinically meaningful pharmacokinetic and therapeutic profile. The current literature therefore represents an important experimental foundation, but substantial preclinical validation remains necessary before hydrogel-based antipsychotic delivery systems can progress from formulation proof-of-concept to clinical investigation [80,210] (Figure 2).

## 8. Conclusions

Hydrogel-based drug-delivery systems expand the formulation possibilities available for sustained psychiatric pharmacotherapy beyond conventional oil depots, crystalline suspensions, and biodegradable microsphere technologies. Their high water content, tunable network architecture, adaptable degradation behavior, and compatibility with injectable, in situ-forming, intranasal, and transdermal approaches provide considerable flexibility for controlling drug loading, administration, and release. The studies identified in this review demonstrate that these principles can be applied to several antipsychotic agents, including risperidone, paliperidone and paliperidone palmitate, aripiprazole, olanzapine, and quetiapine, as well as the experimental antipsychotic peptide PAOPA. Nevertheless, the maturity of the evidence varies substantially among drugs and formulations.

The next phase of development should therefore focus less on demonstrating that hydrogels can prolong drug release and more on determining whether they can do so reproducibly, safely, and with a pharmacokinetic profile that offers a genuine advantage over existing LAIs. This requires integration of material characterization with pharmacokinetics, pharmacodynamics, behavioral efficacy, injection-site tolerability, degradation, repeated-dose safety, and appropriately selected large-animal studies. Particular attention should be directed toward control of burst release, reproducibility of depot formation, sterilization and manufacturing scalability, long-term storage stability, and the relationship between polymer degradation and both local tissue response and systemic exposure.

Hydrogels should therefore be regarded not as replacements for all existing depot technologies, but as a versatile platform from which specific long-acting products may be engineered according to the pharmacological and clinical requirements of individual antipsychotic agents. The current literature provides a convincing pharmaceutical proof-of-concept, but not yet sufficient evidence for broad clinical implementation. Progress toward translation will depend on moving from formulation-centered studies toward integrated, standardized preclinical programmes capable of demonstrating controlled exposure, sustained therapeutic activity, biocompatibility, and clinically relevant advantages over established long-acting antipsychotic formulations, followed by well-designed clinical trials evaluating safety, pharmacokinetics, therapeutic efficacy, and patient acceptability.

## Figures and Tables

**Table 1:** Representative conventional long-acting antipsychotic formulations and their drug-release characteristics.

Antipsychotic/Formulation	Formulation Technology	Carrier/Vehicle	Principal Basis of Prolonged Release	Relevant Consideration	Ref.
Haloperidol decanoate	Oil-based depot solution	Sesame oil	Lipophilic decanoate ester is slowly released from the oil depot and subsequently hydrolyzed to active haloperidol.	Butyrophenone. Release and absorption depend on partitioning from the oil depot and ester hydrolysis. Local injection-site reactions may occur.	[52,53,55,56]
Fluphenazine decanoate	Oil-based depot solution	Sesame oil	Slow release of the lipophilic ester from the oil phase followed by hydrolysis to fluphenazine.	Piperazine phenothiazine compound. Oil-based administration. Injection-site discomfort/local reactions are possible.	[54,55,56]
Flupentixol decanoate	Oil-based depot solution	Low-viscosity vegetable oil (fractionated coconut oil)	Slow release of decanoate ester from oil depot followed by hydrolysis to active drug.	Thioxanthine antipsychotic—Lipophilic ester/oil-depot system.	[55,56]
Zuclopenthixol decanoate	Oil-based depot solution	Thin vegetable oil (fractionated coconut oil)	Slow release from oil depot followed by ester hydrolysis.	Thioxanthine compound. Similar oil-depot principle to other first-generation decanoate LAIs.	[55,56]
Olanzapine pamoate	Microcrystalline aqueous suspension	Aqueous vehicle	Slow dissolution of microcrystalline olanzapine pamoate at the injection site followed by dissociation to olanzapine.	Olanzapine pamoate dissociates readily in aqueous environments (e.g., plasma), so its pharmacokinetic properties depend on the method of administration.	[38,55,56]
Paliperidone palmitate	Aqueous nanocrystal/nanosuspension	Aqueous vehicle	Slowly dissolving palmitate ester particles are hydrolyzed to active paliperidone after dissolution.	Paliperidone (9-hydroxy-risperidone) is an active metabolite of risperidone.	[38,55,56]
Aripiprazole monohydrate	Lyophilized powder	Aqueous vehicle	Slowly absorbed into systemic circulation because of its low solubility.	Requires control of particle characteristics and dissolution. Initiation regimen is formulation-specific.	[38]
Risperidone	PLGA polymeric microspheres	Aqueous suspension	Small initial diffusion phase, 3-week lag and major release associated with increasing PLGA porosity/degradation and erosion.	Delayed main release necessitates oral antipsychotic supplementation during the first 3 weeks.	[55,56]

**Table 3:** Rodent models of schizophrenia with potential application in the preclinical evaluation of hydrogel-based antipsychotic delivery systems.

Model Category	Experimental Model	Strengths and Main Schizophrenia-Relevant Phenotype(s)	Limitations	Ref.
Pharmacological—dopaminergic	Amphetamine/methamphetamine model	-experimentally simple and relatively rapid; -hyperlocomotion and stereotypy; -sensorimotor gating deficits measured in prepulse inhibition (PPI); -latent inhibition deficits.	-limited validity for negative symptoms, particularly social withdrawal; -inconsistent representation of cognitive impairment; -hyperlocomotion is an indirect and non-specific proxy for human psychosis; -behavioral outcomes are highly dependent on dose.	[121,129,130,131]
Pharmacological—dopaminergic	Apomorphine model	-rapid, simple and highly reproducible dopaminergic challenge -stereotypy; -climbing; -locomotor activation.	-low construct validity for schizophrenia because the model directly stimulates dopamine receptors rather than reproducing disease pathophysiology.	[150,151,152,153]
Pharmacological— glutamatergic	Ketamine	-simple, inexpensive and rapidly inducible; -comparatively strong face, construct and predictive validity; -hyperlocomotion; -altered social behavior; -NMDA-receptor hypofunction has mechanistic relevance to schizophrenia and ketamine can generate positive-like, negative-like and cognitive abnormalities; -impairments in hippocampal memory, working memory, and fear learning.	-vary widely in terms of dose, route, frequency, and duration; -high doses may cause sedation, anesthesia or motor impairment that confound behavioral testing.	[121,132,133,134,135,136]
Pharmacological— glutamatergic	Phencyclidine (PCP)	-a very simple, cheap, and not time-consuming process; -hyperlocomotion; -social withdrawal; -impaired PPI; -cognitive deficits.	-after PCP administration, animals are very excited and may be aggressive; -limited value in explaining the etiopathology of the illness.	[121,132,154,155]
Pharmacological— glutamatergic	MK-801/dizocilpine	-relatively cheap and easy-to-perform model; -noncompetitive NMDA receptor antagonist with great inhibitory potency; -working perturbations; -hyperlocomotion; -deficits in PPI; -lowered sociability; -fear conditioning perturbations.	-MK-801-induced behavioral alterations are more representative of an acute psychotic episode than of the persistent and complex phenotype of schizophrenia.	[83,121,156,157,158]
Neurodevelopmental	Gestational methylazoxymethanol acetate (MAM) exposure	-selective effect on developing paralimbic, frontal, and temporal cortices, regions where deficits are typically observed in schizophrenia patients; -increased dopamine neuron population activity; -prepulse inhibition of startle; -latent inhibition; -deficits in working memory tasks; -hyper-responsivity to psychomotor stimulants; -decreased social interaction.	-MAM is toxic and carcinogenic, requiring strict safety precautions; -accurate gestational timing is essential, and intraperitoneal administration carries a risk of miscarriage.	[121,137,138,139]
Neurodevelopmental	Neonatal ventral hippocampal lesion (NVHL) model	-reproduces a broad spectrum of schizophrenia-relevant anatomical, histological, physiological, and behavioral abnormalities; -impaired sensorimotor gating, social interactions, attention, working memory, spatial memory; -hyperlocomotion; -hypersensitivity to psychostimulants; -enhanced addictive behavior, such as increased self-administration of cocaine, nicotine, alcohol, and methamphetamine.	-laborious and technically demanding; -requiring precise neonatal surgery with an increased risk of anesthesia- and procedure-related mortality.	[121,140,141,142,143]
Neurodevelopmental	Maternal immune activation (MIA) model: administration of polyinosinic:polycytidylic acid (poly(I:C)), or lipopolysaccharides (LPS) during the gestational stage	-produces progressive anatomical, histological, physiological, and behavioral abnormalities relevant to schizophrenia, emerging prenatally and becoming evident after puberty, thereby resembling the neurodevelopmental course of the disorder.	-highly sensitive to the immune stimulant (poly(I:C) or LPS), dose, gestational timing, maternal immune response; -risk of miscarriage.	[121,159,160,161]
Environmental/ Neurodevelopmental	Post-weaning social isolation	-highly adaptable and suitable for combination with pharmacological or genetic manipulations to generate more robust, multifactorial schizophrenia-relevant phenotypes.	-limited specificity when used alone and often requires combination with additional pharmacological, genetic, or environmental manipulations to generate a more robust schizophrenia-relevant phenotype (e.g., ketamine, NMDA receptor antagonist); -highly sensitive to the timing and duration of isolation, and housing conditions; -may also induce stress-, anxiety-, and depression-related phenotypes.	[162,163,164,165]
Genetic	Disrupted-in-Schizophrenia 1 (DISC1)	-strong construct validity for studying DISC1-related neurodevelopmental mechanisms, reproducing schizophrenia-relevant morphological, neurophysiological, cognitive, and behavioral abnormalities.	-expensive and time-consuming to generate; -limited by the polygenic nature of schizophrenia and the controversial association between DISC1 and the disorder.	[121,144,145]
Genetic	Deletion in the 22q11.2 Region Model	-strong genetic and construct validity, as 22q11.2 deletion is a major genetic risk factor for schizophrenia; -reproduces schizophrenia-relevant synaptic, circuit, sensorimotor-gating, social, and cognitive abnormalities.	-expensive and technically complex; -phenotypes vary with deletion size; -limited disease specificity, as 22q11.2 deletion is also associated with other neurodevelopmental and neurological disorders such as parkinsonism, autism.	[121,146,147,148]
Genetic	Dysbindin-1 Model	-reproduces schizophrenia-relevant dopaminergic, glutamatergic, and GABAergic dysfunction, together with social and cognitive deficits.	-limited by its single-gene nature and strong dependence on genetic background; -show poor suitability for PPI assessment and may display behavioral phenotypes inconsistent with other schizophrenia models.	[121,149,166]
Genetic	Neurotrophic Factor Neuregulin 1 (NRG1) Model	-strong construct validity for investigating NRG1–ErbB4 signaling, reproducing schizophrenia-relevant alterations in excitatory/inhibitory balance, NMDA-receptor function, GABAergic transmission, and behavioral phenotypes.	-expensive, technically complex, and time-consuming to generate; -phenotype depends on the specific genetic manipulation, while targeting a single signaling pathway cannot reproduce the complex polygenic nature of schizophrenia.	[121,167,168,169]

**Table 4:** Behavioral tests relevant to the preclinical evaluation of antipsychotic efficacy and schizophrenia-related phenotypes in rodents.

Test	Domain	Test Description/Parameters Assessed	Utility in the Experimental Context	Ref.
Social interaction test	Negative-like/social function	Rats were placed in an open arena for 5 min. Total interaction time was recorded and divided into active interaction (sniffing, following, crawling over/under, grooming, aggressive behavior) and passive interaction (close proximity).	Reduced social interaction was used as a behavioral correlate of negative schizophrenia-like symptoms and to assess the ability of intranasal PAOPA-loaded SNP-CMCh hydrogel to reverse MK-801-induced social deficits.	[83]
Forced swimming test (FST)	Negative schizophrenia-like symptoms	Mice were placed in water for 6 min. After a 2-min habituation period, immobility, swimming, and climbing times were measured during the final 4 min.	Used by the authors to evaluate the effects of treatment on negative schizophrenia-like symptoms induced by ketamine.	[99]
Sucrose preference test (SPT)	Negative schizophrenia-like symptoms/anhedonia	Rodents are given a choice between a sucrose solution and water. Sucrose preference is calculated as the proportion of sucrose solution consumed relative to total fluid intake.	Used to assess anhedonia/reward-related deficits as a behavioral correlate of negative schizophrenia-like symptoms. Reduced sucrose preference indicates impaired hedonic/reward-related behavior.	[172,173,174]
Modified tail suspension test	Despair as a characteristic of the negative symptoms of schizophrenia	Rats were suspended by the tail for 5 min and immobility duration was recorded. Repeated rotation, twisting, and body-lifting attempts represented increased mobility.	Used to assess behavioral despair and lack of motivation and their modification by antipsychotic treatment in the schizophrenia-like model.	[92]
Locomotor activity test	Positive schizophrenia-like symptoms	Locomotor activity was measured in an arena using photobeam interruptions generated by animal movement and rearing. Increased locomotor activity denotes positive symptoms in schizophrenia.	Ketamine-induced hyperlocomotion was considered a correlate of positive schizophrenia-like symptoms.	[31,99]
Open field test (OFT)	Positive/negative schizophrenia-like symptoms	Exploration and emotionality were evaluated using latency, number of squares crossed, rearing frequency, and grooming frequency during a 5-min session.	Used to characterize ketamine-induced behavioral abnormalities and treatment response. The authors relate increased exploratory activity to positive symptoms and altered emotionality to negative symptoms.	[92]
Morris water maze (MWM)	Spatial learning and memory	Rats were trained to locate a submerged platform. The study measured escape latency, time spent in the target quadrant, and path length/total distance travelled during the task.	Used to evaluate spatial learning and memory impairment induced by MK-801 and determine whether sustained aripiprazole delivery preserved neurocognitive performance.	[175]
Novel object recognition test (NORT)	Cognitive symptoms/recognition memory	Rodents are first exposed to two identical objects and, after a retention interval, one object is replaced by a novel object. Exploration time of the novel versus familiar object, usually expressed as a discrimination or recognition index, is recorded.	Used to assess recognition memory and cognitive deficits relevant to schizophrenia. A reduced preference for the novel object indicates impaired recognition memory, while restoration of novel-object preference can indicate improvement following antipsychotic treatment.	[176,177]
Five-choice serial reaction time task (5-CSRTT)	Cognitive symptoms/attention and inhibitory control	Rodents are trained to detect and respond to a brief visual stimulus presented unpredictably in one of five spatial locations. Main parameters include response accuracy, omissions, premature and perseverative responses, and response latency.	Used to assess visuospatial attentional deficits and impaired inhibitory control relevant to cognitive dysfunction in schizophrenia, and to evaluate their modulation by antipsychotic treatment.	[174,178]
Attentional set-shifting task (ASST)	Executive function and cognitive flexibility	Rodents learn to discriminate between stimuli based on different dimensions, typically odor and digging medium, to obtain a food reward. Performance across discrimination, reversal, intra-dimensional, and extra-dimensional shift stages is assessed, commonly by the number of trials required to reach criterion.	Used to assess cognitive flexibility, rule shifting, and executive-function deficits relevant to schizophrenia.	[179,180,181]
Y-maze test	Spontaneous alternation behavior/working memory	Rats freely explored three arms for 5 min. Spontaneous alternation percentage was calculated from consecutive entries into all three arms.	Used to evaluate spontaneous alternation and cognitive performance, allowing assessment of ketamine-induced cognitive deficits and their reversal by sustained risperidone delivery.	[92]
Bar test	Measurement of catalapetic adverse effects of antipsychotic drugs	Rats’ forepaws were positioned on an elevated horizontal bar and latency to remove one or both forepaws was recorded.	The potential of risperidone to increase the immobility duration on bar as a parameter of catalepsy.	[92]
Prepulse Inhibition (PPI)	Sensorimotor gating	PPI measures the reduction in the startle response when a weak, non-startling acoustic prepulse is presented shortly before a strong startling stimulus.	Induced animal models of schizophrenia have revealed a deficit in PPI.	[130,174,182]

## Data Availability

No new data were created or analyzed in this study. Data sharing is not applicable to this article.

## Abbreviations

The following abbreviations are used in this manuscript:

3D	Three-dimensional
5-CSRTT	Five-choice serial reaction time task
βGP	β-glycerol phosphate
APZ	Aripiprazole
ASST	Attentional set-shifting task
BSIFG	Bio-shielding in situ-forming gel
cHA	Cross-linked hyaluronic acid
CMCh	Carboxymethyl chitosan
COL	Chitosan oligosaccharide lactate
CUMS	Chronic unpredictable mild stress
DISC1	Disrupted-in-Schizophrenia 1
FST	Forced swimming test
GA	Glutaraldehyde
H&E	Hematoxylin and eosin
HFMAPs	Hydrogel-forming microneedle array patches
HG-LCN	Lipid-core nanocapsules in hydrogel
HP-β-CD	Hydroxypropyl-β-cyclodextrin
HPMC	Hydroxypropyl methyl cellulose
ISFI	In situ-forming implant
ISGFI	In situ gel-forming implant
LAI	Long-acting injectable
LPS	Lipopolysaccharides
MAM	Methylazoxymethanol acetate
MIA	Maternal immune activation
MK-801	((+)-5-methyl-10,11-dihydro-5H-dibenzo [a,d]cyclohepten-5,10-imine maleate salt)
MN	Microneedle
MWM	Morris water maze
NHPs	Non-human primates
NMDA	N-methyl-D-aspartate
NNH	Nanoparticle network hydrogel
NORT	Novel object recognition test
NRG1	Neurotrophic Factor Neuregulin 1
NVHL	Neonatal ventral hippocampal lesion
OFT	Open field test
OLZ	Olanzapine
PAOPA	(3(R)-[(2(S)-pyrrolidinylcarbonyl)amino]-2-oxo-1-pyrrolidineacetamide)
PBAP	Phenylboronic acid pinacol ester
PCP	Phencyclidine
PLGA	Poly (lactic-co-glycolic acid)
PLPD	Paliperidone
PNAGA	Poly (N-acryloyl glycinamide)
poly(I:C)	Polyinosinic:polycytidylic acid
PPI	Prepulse inhibition
PPP	Paliperidone palmitate
QTP	Quetiapine
QTP-F	Quetiapine fumarate
RDPA	R6-modified, ammonia borane-loaded PBAP–dextran nanoparticles
RIS	Risperidone
SNPs	Starch nanoparticles
SPT	Sucrose preference test
